# Association of CYP2A6 gene deletion with cancers in Japanese elderly: an autopsy study

**DOI:** 10.1186/s12885-020-6663-4

**Published:** 2020-03-04

**Authors:** Maidina Abudushataer, Noriko Sato, Makiko Mieno, Motoji Sawabe, Masaaki Muramatsu, Tomio Arai

**Affiliations:** 1 Department of Molecular Epidemiology, Medical Research Institute, Tokyo Medical and Dental University, Tokyo, Japan; 2 Center for Information, Jichi Medical University, Tochigi, Japan; 3 Department of Molecular Pathology, Graduate School of Medical and Dental Sciences, Tokyo Medical and Dental University, Tokyo, Japan; 4 Department of Pathology, Tokyo Metropolitan Geriatric Hospital, Tokyo, Japan

**Keywords:** CYP2A6*1, CYP2A6*4, Pathology, Cancer, Smoking

## Abstract

**Background:**

CYP2A6 is an enzyme involved in oxidation of a number of environmental chemicals, including nicotine, pro-carcinogenic nitrosamines and polycyclic aromatic hydrocarbons (PAHs). The whole gene deletion of CYP2A6 (CYP2A6*4) is prevalent in East Asian population. Whether or not CYP2A6*4 associates with cancer is still controversial.

**Methods:**

We undertook an association study to determine whether deletion of CYP2A6 gene associates with total cancer and major cancer types employing data of consecutive autopsy cases registered in the Japanese single-nucleotide polymorphisms for geriatric research (JG-SNP) database. The presence of cancer were inspected at the time of autopsy and pathologically confirmed. Genotyping for CYP2A6 wild type (W) and deletion (D) was done by allele specific RT-PCR method.

**Results:**

Among 1373 subjects, 826 subjects (60.2%) were cancer positive and 547 subjects (39.8%) were cancer negative. The genotype frequency in the whole study group for WW, WD and DD were 65.0, 30.6 and 4.4%, respectively, which obeyed the Hardy-Weinberg equilibrium (*p* = 0.20). Total cancer presence, as well as major cancers including gastric, lung, colorectal, and blood cancers did not show any positive association with CYP2A6 deletion. When male and female were separately analyzed, CYP2A6 deletion associated with decreased gastric cancer risk in female (OR = 0.49, 95%CI: 0.25–0.95, *p* = 0.021, after adjustment for age, smoking and drinking). When smoker and non-smoker were separately analyzed, CYP2A6 deletion associated with decreased total cancer in female nonsmokers (OR = 0.67, 95%CI: 0.45–0.99, *p* = 0.041 after adjustment). On the other hand, CYP2A6 deletion associated increase blood cancers in smokers (OR = 2.05, 95%CI: 1.19–3.53, *p* = 0.01 after adjustment).

**Conclusion:**

The CYP2A6 deletion may not grossly affect total cancer. It may associate with individual cancers in sex and smoking dependent manner. Further studies with larger sample size are warranted to confirm our results.

## Background

Cytochrome P450 2A6 (CYP2A6) is known as a pharmacogene, primarily expressed in the liver, and catalyzes several clinically used drugs, including tegafur, letrozole, caffeine and other substrates [1 ,2]. CYP2A6 plays a major role in the nicotine metabolic pathway. It catalyzes nicotine to inactive metabolic cotinine, followed by further metabolism of cotinine to trans-3′-hydroxycotinine (3HC) [2, 3]. CYP2A6 is also responsible for the metabolic activation of tobacco-specific pro-carcinogens such as 4-(methylnitrosamino)-1-(3-pyridyl)-1-butanone (NNK) and 4-(methylnitrosamino)- 1-(3-pyridyl)-1-butanol (NNAL), which are found in the cigarette smoke [4 ,5]. Recently, CYP2A6 enzyme has been shown to metabolize not only tobacco-related nitrosamines but also polycyclic aromatic hydrocarbons (PAHs) and aryl- and heterocyclic amines [6].

CYP2A6 gene is highly polymorphic, with more than 40 variants identified to date [7]. Genetic variations of CYP2A6 gene have been widely studied with respect to its association with levels of nicotine metabolites in vivo, nicotine dependence, smoking behavior and cancer susceptibility. The allele frequencies of CYP2A6 genetic variants differ by racial/ethnic groups. Wild type allele CYP2A6*1 is considered as a wild type reference allele. In comparison, the whole gene deletion allele CYP2A6*4 is one of the most common variants in Asian ancestry, with MAF of 20~24% in Japanese [8, 9, 10, 11]. The CYP2A6 deletion has been studied with respect to various tobacco-related cancers, but there remain inconsistent results. To this end, we employed consecutive autopsy cases of Japanese elderly registered in the JG-SNP database to determine the association between CYP2A6 deletion and risk of total cancer and major cancer types. This was thought to be important since recent haplotype analysis of CYP2A6 gene revealed that CYP2A6*4 cannot be tagged by nearby SNPs (r^2^ < 0.4) [12].

## Methods

### Subjects

The study population was obtained from Internet database of Japanese single-nucleotide polymorphisms (SNPs) for geriatric research (JG-SNP) [13]. Briefly, JG-SNP database consists of consecutive autopsy cases performed at the Tokyo Metropolitan Geriatric Hospital between 1995 and 2006. Pathological diagnosis was made by MS and TA. Clinical information including subject age, gender, smoking and drinking history was obtained from medical chart. Informed consent for the use of autopsy samples and patient clinical data for this study was obtained from the family of study participants at the time of autopsy. The definition of the drinkers/smokers were defined by current drinkers/smokers and ever drinkers/smokers. Never drinkers/smokers were those who never drink/smoke. Clinical data such as drinking and smoking habit were extracted from patient medical records. The study protocol was approved by the Ethics Committees of Tokyo Geriatric Hospital and Tokyo Medical and Dental University and authorized by TMDU Research Ethics Committee under the number 2016–011-02.

The genomic DNA was isolated from kidney cortex by phenol-chloroform method and stored at − 20 °C until use. Concentration of DNA samples was determined by optical density of 260/280. We used 1373 subjects of which the DNA samples were present.

### Primer/probe design and reaction mixture

The primer and probe sets were designed to detect the presence and absence of CYP2A6 gene as previously described by Yamazaki et al. [14]. The sequence of primers and probes are shown in Table 1. The reaction was performed in a 10 μL volume with 10 ng genomic DNA. The reaction mixture contained 0.75 μM CYP2A6_ex2_3_F, 1.0 μM CYP2A7_ex9_F, 0.25 μM CYP2A6_ex2_3_R, 0.25 μM CYP2A6_3UTR_R, 0.25 μM CYP2A6_ex2_3_probe, 0.4 μM CYP2A6_4_probe and TaqMan GTXpress™ Master Mix (Applied Biosystems). CYP2A6_ex2_3_probe was labeled with hexachloro-fluorescein (HEX) for detecting CYP2A6 wild-type, and CYP2A6_4_probe was labeled with 6-fluorescein amidite (FAM) for detecting CYP2A6 whole-gene deletion.

**Table 1:** Primer and probe sets for detecting CYP2A6*1 and *4 allele

	Primers and probes	Target Sequence
CYP2A6*1	Forward Primer (CYP2A6_ex2_3_F)	5′-AGCTCTGCTGGGCAA-3′
	Reverse Primer (CYP2A6_ex2_3_R)	5′-CCCCTGCTCACCGCCA-3′
	Probe (CYP2A6_ex2_3_probe)	5′-TGTCTCCATTCCCGCGTTCA-3′(HEX)
CYP2A6*4	Forward Primer (CYP2A7_ex9_F)	5′-TCCCCCAAACACGTGGT-3′
	Reverse Primer (CYP2A6_3UTR_R)	5′-AGGTGAGCGTGCAATG-3′
	Probe (CYP2A6_4_probe)	5′-AGAGGGAAGAGAAGAAACAGAA-3′(FAM)

### Quantitative real-time PCR

The genotyping for the CYP2A6 gene was done by using multiplex real-time polymerase chain reaction (RT-PCR) method with the above dual-labeled probes. The genomic DNAs were placed into 384 well plates, and were dried down. Mixture of above reagent was added to each well, centrifuged briefly, then sealed by foil and the PCR reaction was run on Light Cycler® 480 I 384 (Roche, Penzberg, Germany). The thermal cycler conditions were initiated at 95 °C for 20 s followed by 40 cycles of denaturation at 95 °C for 20 s, annealing at 60 °C for 30 s and extension at 72 °C for 31 s. The genotype was determined from RT-PCR fluorescent curve by optical observation.

### Statistical analysis

Hardy-Weinberg Equilibrium (HWE) for CYP2A6 genotypes was determined using Chi-Square test. Chi- Square or Fisher’s exact tests were used to compare CYP2A6 genotypes and cancer or behavior habits of smoking and drinking. Adjustments were done for age, sex, smoking and drinking status where appropriate. Binominal logistic regression was used to calculate the odds ratio (OR), 95% confidence-intervals (CI) and the p-values to estimate the relationship. All p-values are reported two-sided, and < 0.05 were considered to be statistically significant. Subgroup analyses based on gender and smoking status were also performed. Adjustment for multiple testing, such as Bonferroni correction, was not applied for its conservative nature. Statistical analysis was performed using the IBM SPSS Statistics software 25.0 (IBM; New York, USA).

## Results

### Characteristics of the study subjects

Selected demographic variables, including the sites of cancers, and risk factors are shown in Table 2. The study included 1373 samples, including 743 men and 630 women. The mean age of subjects was 80.1 ± 9.0 years. The subjects consist of 655 smokers and 633 non-smokers with 85 missing data. Alcohol drinking data include 456 drinkers and 833 non-drinkers and 84 missing data. Among all samples, 826 patients were diagnosed as having at least one cancer (60.2%), while others were cancer negative (*n* = 547, 39.8%). The major cancer includes gastric cancer (*n* = 163, 11.9%), colorectal cancer (*n* = 130, 9.5%), lung cancer (*n* = 128, 9.3%), and blood cancer (*n* = 127, 9.3%). In this study, we evaluated these high prevalent cancers.

**Table 2:** Distribution of selected demographic variables and risk factors

	All subjects (*n* = 1373)	Cancer-free (n = 547)	Cancer-bearing (*n* = 826)	p-value*
Age at death, years†	80.1 ± 9.0			
≥ 80、n (%)	729 (53)	300 (55)	429 (52)	
< 80	644 (47)	247 (45)	397 (48)	0.2946
Gender, n (%)				
Male	743 (54)	273 (50)	470 (57)	
Female	630 (46)	274 (50)	356 (43)	**0.0128**
Smoking, n (%)				
Non-smoker	633 (46)	256 (47)	377 (45)	
Smoker	655 (48)	245 (45)	410 (50)	0.2776
Missing	85 (6)	46 (8)	39 (5)	
Alcohol, n (%)				
Non-drinker	833 (61)	346 (63)	487 (59)	
Drinker	456 (33)	157 (29)	299 (36)	**0.0143**
Missing	84 (6)	44 (8)	40 (5)	
Cancer, n (%)				
0			547 (40)	
1			604 (44)	
2			178 (13)	
≥ 3			44 (3)	
Cancer sites, n (%)				
Gastric cancer			163 (12)	
colorectal cancer			130 (10)	
Lung cancer			128 (9)	
Blood cancer			127 (9)	

^†^ means ± SDs

^*^p-value for Fisher’s exact probability test

### Genotyping and allele frequency of CYP2A6

**Fig. 1:**
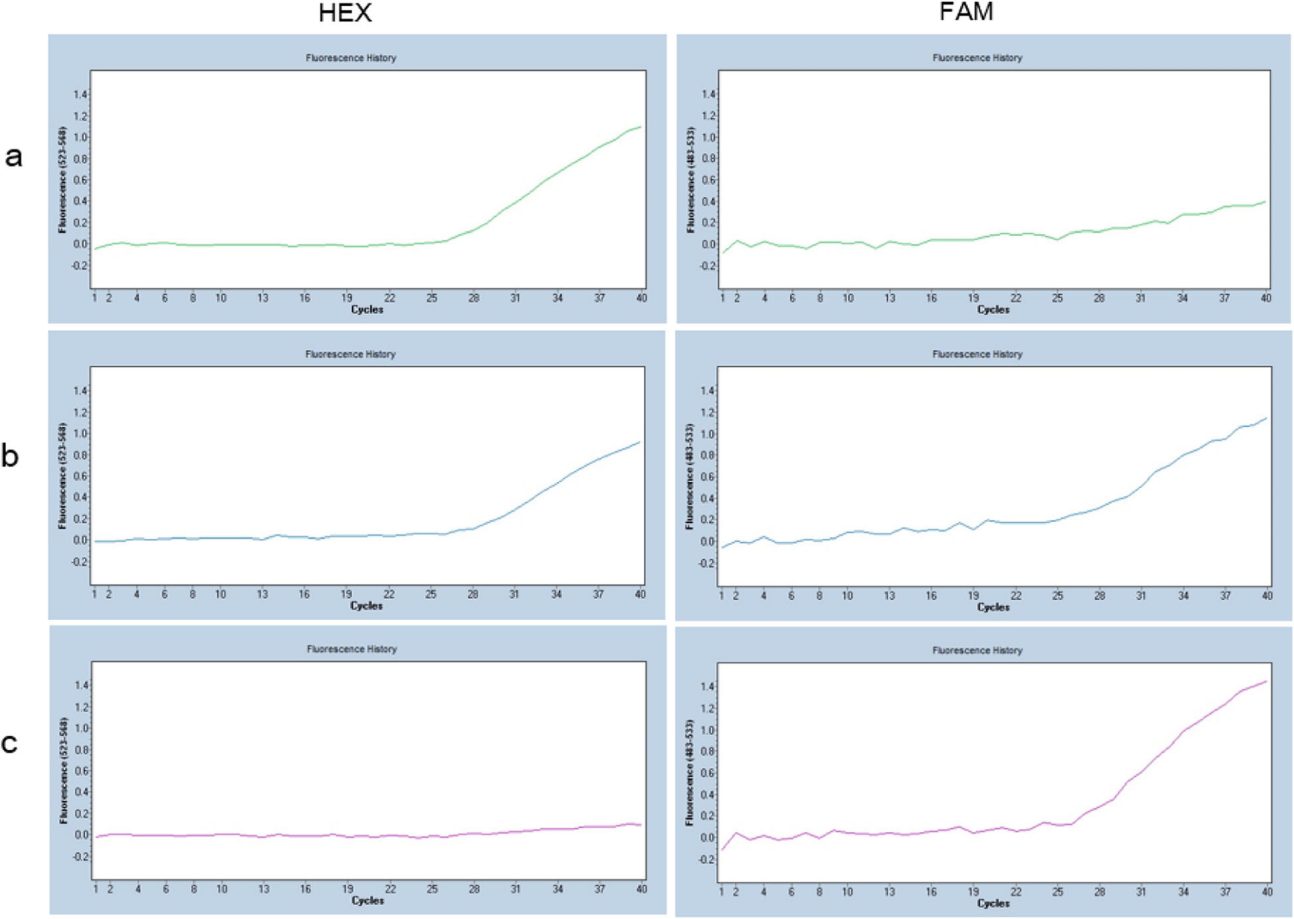
Typical RT-PCR results of CYP2A6 genotyping for CYP2A6*1/*1 (**a**), CYP2A6*1/*4 (**b**), and CYP2A6*4/*4 (**c**)

A typical reaction curves are shown in Fig. 1. Based on the obtained amplification curves of HEX and FAM fluorescence, genotypes of CYP2A6 wild type homozygotes, heterozygotes and deletion homozygotes were determined. The presence of CYP2A6 (W) showed HEX fluorescence but did not show FAM fluorescence (Fig. 1.a). The heterozygote CYP2A6 W and deletion (D) showed both fluorescences (Fig. 1.b). Whole-gene deletion type CYP2A6 (D) showed FAM fluorescence but did not show HEX fluorescence (Fig. 1.c). Among all samples, 892 subjects were determined as W/W (65.0%), 420 samples were determined as W/D (30.6%) and 61 samples were determined as D/D (4.4%). The allele frequencies of CYP2A6 W and D alleles were 80.2 and 19.8%, respectively. The whole study group obeyed the HWE (*p* = 0.20), and the allele frequency was consistent with previous studies in the Japanese population [8, 9, 10, 11].

### Association of CYP2A6 genotype with cancer

First, association of CYP2A6 W/D genotype and total cancer were determined for all subjects (Table 3). We studied the association of CYP2A6 (D) and total cancer in different models of dominant (DD + WD: WW), recessive (DD: WD + WW) and additive (WW: WD: DD). In any of the genetic models, no association was found in crude analysis and after adjusted for age, gender, smoking and drinking. The genotype distribution obeyed HWE in cancer-free group (*p* = 0.57), but slightly deviated in cancer-bearing group (*p* = 0.03). Association for independent cancer of gastric, colorectal, lung and blood were also studied and no associations were found.

**Table 3:** Genetic models for total cancer

Dominant		WW (%)	WD + DD (%)		p-value*	p-value**
	–	343(63)	204(37)		0.153	0.132
	+	549(66)	277(34)			
Recessive		WW + WD (%)		DD (%)		
	–	526(96)		21(4)	0.377	0.539
	+	786(95)		40(5)		
Additive		WW (%)	WD (%)	DD (%)		
	–	343(63)	183(33)	21(4)	0.142	
	+	549(66)	237(29)	40(5)		

*WW* wild-type (reference); *WD* heterozygote; *DD* whole-gene deletion;-(cancer-free); + (cancer-bearing)

^*^p-value was done by Crude analysis

^**^ Logistic regression analysis adjusted by age, gender, smoking and drinking

Next, we separately analyzed male and female (Table 4). In male, association of CYP2A6 W/D genotypes gave no association for total cancer, and independent cancers in crude analysis and adjusted for age, smoking and drinking. As for blood cancer there was a positive sign of association (WD + DD/WW, OR = 1.72, 95%CI: 1.07~2.75, *p* = 0.024) in crude analysis, which did not remain positive after adjustment (*p* = 0.063).

**Table 4:** Association between CYP2A6*4 and risk of developing cancer

		Total subjects				Male				Female			
		WD + DD/WW (%)	OR(95%CI)	*P*	P*	WD + DD/WW (%)	OR(95%CI)	*P*	P**	WD + DD/WW (%)	OR(95%CI)	*P*	P**
Total CA(*n* = 1373)	–	204(37)/343(63)	0.85 (0.68–1.06)	0.153	0.132	92(34)/181(66)	1.00 (0.73–1.37)	0.982	0.772	112(41)/162(59)	0.73 (0.52–1.01)	0.055	0.053
	+	277(34)/549(66)				158(34)/312(66)				119(33)/237(67)			
Gastric CA(*n* = 1372)	–	429(35)/780(65)	0.85 (0.60–1.21)	0.369	0.280	210(33)/422(67)	1.13 (0.74–1.73)	0.564	0.735	219(38)/358(62)	0.49 (0.25–0.95)	**0.036**	**0.021**
	+	52(32)/111(68)				40(36)/71(64)				12(23)/40(77)			
Colorectal CA(*n* = 1372)	–	438(35)/804(65)	0.91 (0.62–1.33)	0.619	0.716	228(34)/447(66)	0.94 (0.55–1.60)	0.813	0.772	210(37)/357(63)	0.87 (0.50–1.51)	0.624	0.818
	+	43(33)/87(67)				22(32)/46(68)				21(34)/41(66)			
Lung CA(*n* = 1370)	–	429(35)/813(65)	1.30 (0.89–1.88)	0.171	0.097	217(33)/443(67)	1.35 (0.84–2.15)	0.212	0.178	212(36)/370(64)	1.28 (0.69–2.36)	0.438	0.322
	+	52(41)/76(59)				33(40)/50(60)				19(42)/26(58)			
Blood CA(*n* = 1371)	–	428(34)/816(66)	1.37 (0.94–1.98)	0.100	0.244	214(32)/449(68)	1.72 (1.07–2.75)	**0.024**	0.063	214(37)/367(63)	0.97 (0.52–1.80)	0.928	0.576
	+	53(42)/74(58)				36(45)/44(55)				17(36)/30(64)			
Smoking (*n* = 1288)	–	223(35)/410(65)	1.00 (0.79–1.25)	0.966		63(34)/122(66)	1.03 (0.73–1.47)	0.853		160(36)/288(64)	1.03 (0.69–1.53)	0.902	
	+	230(35)/425(65)				181(35)/339(65)				49(36)/86(64)			
Alcohol (*n* = 1289)	–	283(34)/550(66)	1.16 (0.91–1.47)	0.235		105(33)/212(67)	1.15 (0.84–1.58)	0.373		178(34)/338(66)	1.41 (0.85–2.36)	0.188	
	+	170(37)/286(63)				141(36)/247(64)				29(43)/39(57)			

*CA* cancer; *WW* wild-type (reference); *WD* heterozygote; *DD* whole-gene deletion;-(cancer-free); + (cancer-bearing)

^*^ Represents adjusted by age, gender, smoking and drinking

^**^ Represents adjusted by age, smoking and drinking

In female, CYP2A6 deletion barely did not reach significant level with total cancer in crude analysis (*p* = 0.055). With respect to colorectal cancer, lung cancer and blood cancer in female, there was no significant association related with CYP2A6 deletion in crude and adjusted analysis. Only, CYP2A6 deletion significantly associated with decreased risk of gastric cancer presence in crude analysis (*p* = 0.036), and remained positive after adjustment (*p* = 0.021). We also determined whether CYP2A6 deletion associates with smoking and drinking habits. As shown in the last two rows of Table 4, these health behaviors were not affected by CYP2A6 deletion.

We further analyzed smoker and nonsmoker (Supplementary Table 1 and 2). As for smoker, CYP2A6 deletion did not associate with total cancer, nor independent cancers of gastric, colorectal and lung. However, there was a positive sign with increased blood cancer in smokers (OR = 2.05, 95%CI: 1.19~3.53, *p* = 0.01, after adjustment). As for non-smokers, total cancer was barely non-significant (OR = 0.73, 95%CI: 0.53–1.02 *p* = 0.08, after adjustment) and also non-significant for independent cancers. Only in female nonsmokers, CYP2A6 deletion associated with decreased total cancer (OR = 0.67, 95%CI: 0.45–0.99, *p* = 0.041 after adjustment).

## Discussion

In this study, we determined the association of CYP2A6 gene deletion genotypes with various cancer risks. Overall, our findings suggested that CYP2A6 deletion do not largely affect presence of total cancer. However, there were some positive signs that CYP2A6 deletion might associate with the presence of independent cancer in sex and smoking dependent manner.

CYP2A6 variation has been recurrently studied for smoking behavior. In general, reduced function of polymorphic CYP2A6 enzyme, including gene deletion has been associated with decreased smoking behaviors [2, 12]. However, our study did not find any association between CYP2A6 deletion and smoking habit (Table 4). This may be due to binominal nature of smoking habits in our database.

CYP2A6 variants have been studied for tobacco-related cancers such as lung [15, 16, 17, 18, 19, 20, 21], bladder [22, 23], esophageal [24], oral [25] and urothelial [26] cancers. Since many cancers are tobacco related, we first studied whether CYP2A6 deletion associate with total cancer in our study sample. We could not find any association for total cancer in dominant, recessive and additive genetic models (Table 3).

We then studied independent cancers of lung, colorectal, gastric, and blood cancers, employing dominant model to detect the association (Table 4). Our results did not find any association between CYP2A6 deletion with any of these cancers. The most studied cancer in the literature is lung cancer and decreased function of CYP2A6 generally associated with lower lung cancer risk [15, 16, 17, 18, 19, 20, 21]^,^.The reason why we could not detect CYP2A6 deletion and lung cancer may be due to small sample size and that we did not consider other low active variants (see the limitation below)**.**

Our study found a significant association between CYP2A6 deletion with decreased risk of gastric cancer in Japanese female. Also CYP2A6 deletion associated with decreased total cancer in non-smoking female. Since CYP2A6 activates not only tobacco-related nitrosamines, but also activate number of environmental chemicals such as polycyclic aromatic hydrocarbons (PAHs) and aryl- and heterocyclic amines, CYP2A6 deletion may have a lower activity to convert them into active carcinogens in vivo.

Intriguingly, CYP2A6 deletion associated with increased blood cancer only in smokers (OR = 2.05, 95%CI: 1.19~3.53, *p* = 0.01, after adjustment). This result is tempting to speculate that the blood cancers might be caused by a different spectrum of carcinogens, which are inactivated by CYP2A6. However, this notion should be considered a working hypothesis and these observations must be replicated in a larger sample size study. Indeed, one Japanese study employing 120 gastric cancer patients and 158 controls has shown that CYP2A6 deletion homozygotes increase gastric cancer risk [27], which appears to be contradictory to our results.

There are limitations in our study. First, this was a hospital-based autopsy study and we had limited access to clinical information on lifestyle variables that could impact the development of cancer. Second, the autopsies were performed on many of the deaths (40%) that occurred in the hospital, and the causes of death among our autopsy cases were similar to those reported in a national survey, we cannot rule out the possibility of selection bias. Such bias can arise from chance of admission, consent to autopsy, cause of death, and autopsy practice. Survival bias is also a possibility, as particular genotypes may be associated with other diseases or influence the lifespan of certain subjects, thereby introducing bias into the study population. Third, the current assay did not discriminate CYP2A6*1 from CYP2A6*7 and *9 which appear to have low activity scores [21]. However, CYP2A6 *4 is the whole gene deletion and expected to have the most extreme phenotype and cannot be tagged by nearby SNPs (r^2^ < 0.4) [12].

## Conclusion

CYP2A6 deletion may not largely affect presence of total cancer. CYP2A6 deletion may associate with decreased gastric cancer in female and decreased total cancer in nonsmoking female. These results warrant further confirmation with larger sample size studies.

## Supplementary information

**Additional file 1.** **Supplementary Table 1.** Association between CYP2A6*4 and risk of developing cancer with dominant model for smokers.

**Additional file 2.** **Supplementary Table 2.** Association between CYP2A6*4 and risk of developing cancer with dominant model for non-smokers.

## Data Availability

All datasets used or analysed for this study are available from the corresponding author upon reasonable request.

## Abbreviations

3HC: Trans-3′-hydroxycotinine
CYP2A6: Cytochrome P450 2A6
FAM: 6-fluorescein amidite
HEX: Hexachloro-fluorescein
HWE: Hardy-Weinberg Equilibrium
JG-SNP: Japanese single-nucleotide polymorphisms for geriatric research
NNAL: 4-(methylnitrosamino)- 1-(3-pyridyl)-1-butanol
NNK: 4-(methylnitrosamino)-1-(3-pyridyl)-1-butanone
PAHs: Polycyclic aromatic hydrocarbons
RT-PCR: real-time polymerase chain reaction
SNPs: Single-nucleotide polymorphisms
