# Supplementary material for: Association of CYP2A6 gene deletion with cancers in Japanese elderly: an autopsy study
Source: BMC Cancer. 2020 Mar 4;20:186. doi: 10.1186/s12885-020-6663-4 (PMC7057549; doi:10.1186/s12885-020-6663-4)
Supplement: Supplementary file 1 — Additional file 1. Supplementary Table 1. Association between CYP2A6*4 and risk of developing cancer with dominant model for smokers. [file 12885_2020_6663_MOESM1_ESM.docx]

Supplementary Table 1 Association between CYP2A6*4 and risk of developing cancer with dominant model for smokers

|  |  | Total subjects (n=655) | | | | Male (n=520) | | | | Female (n=135) | | | |
| --- | --- | --- | --- | --- | --- | --- | --- | --- | --- | --- | --- | --- | --- |
|  |  | WD+DD/WW (%) | OR(95%CI) | *P* | *P** | WD+DD/WW (%) | OR(95%CI) | *P* | *P*** | WD+DD/WW (%) | OR(95%CI) | *P* | *P*** |
| Total CA | - | 88(36)/157(64) | 0.95  (0.67-1.32) | 0.739 | 0.755 | 65(36)/118(64) | 0.95 (0.65-1.39) | 0.802 | 0.817 | 23(37)/39(63) | 0.94 (0.46-1.90) | 0.859 | 0.795 |
|  | + | 142(35)/268(65) |  |  |  | 116(34)/221(66) |  |  |  | 26(36)/47(64) |  |  |  |
| Gastric CA | - | 201(36)/363(64) | 0.85  (0.53-1.36) | 0.485 | 0.449 | 153(35)/288(65) | 1.03 (0.63-1.71) | 0.898 | 0.99 | 48(39)/75(61) | 0.14 (0.02-1.14) | 0.066 | 0.074 |
|  | + | 29(32)/62(68) |  |  |  | 28(35)/51(65) |  |  |  | 1(8)/11(92) |  |  |  |
| Colorectal CA | - | 208(35)/382(65) | 0.94  (0.55-1.61) | 0.821 | 0.916 | 164(35)/303(65) | 0.87 (0.48-1.60) | 0.660 | 0.725 | 44(36)/79(64) | 1.28 (0.38-4.28) | 0.686 | 0.599 |
|  | + | 22(34)/43(66) |  |  |  | 17(32)/36(68) |  |  |  | 5(42)/7(58) |  |  |  |
| Lung CA | - | 194(35)/366(65) | 1.15 (0.73-1.81) | 0.539 | 0.371 | 153(34)/293(66) | 1.17 (0.70-1.94) | 0.555 | 0.413 | 41(36)/73(64) | 1.10 (0.42-2.86) | 0.852 | 0.669 |
|  | + | 36(38)/59(62) |  |  |  | 28(38)/46(62) |  |  |  | 8(38)/13(62) |  |  |  |
| Blood CA | - | 201(34)/397(66) | 2.05  (1.19-3.53) | **0.010** | **0.010** | 155(33)/313(67) | 2.02 (1.13-3.60) | **0.017** | **0.018** | 46(35)/84(65) | 2.74 (0.44-17.09) | 0.279 | 0.363 |
|  | + | 29(51)/28(49) |  |  |  | 26(50)/26(50) |  |  |  | 3(60)/2(40) |  |  |  |
| Alcohol | - | 93(32)/198(68) | 1.30 (0.94-1.81) | 0.113 |  | 62(32)/131(68) | 1.25 (0.85-1.82) | 0.253 |  | 31(32)/67(68) | 1.82 (0.83-4.01) | 0.137 |  |
|  | + | 134(38)/219(62) |  |  |  | 118(50)/200(50) |  |  |  | 16(46)/19(54) |  |  |  |

CA, cancer; WW, wild-type (reference) ; WD, heterozygote; DD, whole-gene deletion;－(cancer-free); + (cancer-bearing).

* Represents adjusted by age, gender and drinking.

** Represents adjusted by age and drinking
