# Supplementary material for: Association of CYP2A6 gene deletion with cancers in Japanese elderly: an autopsy study
Source: BMC Cancer. 2020 Mar 4;20:186. doi: 10.1186/s12885-020-6663-4 (PMC7057549; doi:10.1186/s12885-020-6663-4)
Supplement: Supplementary file 2 — Additional file 2. Supplementary Table 2. Association between CYP2A6*4 and risk of developing cancer with dominant model for non-smokers. [file 12885_2020_6663_MOESM2_ESM.docx]

Supplementary Table 2 Association between CYP2A6*4 and risk of developing cancer with dominant model for non-smokers

|  |  | Total subjects (n=633) | | | | Male (n=185) | | | | Female (n=448) | | | |
| --- | --- | --- | --- | --- | --- | --- | --- | --- | --- | --- | --- | --- | --- |
|  |  | WD+DD/WW (%) | OR(95%CI) | *P* | *P** | WD+DD/WW (%) | OR(95%CI) | *P* | *P*** | WD+DD/WW (%) | OR(95%CI) | *P* | *P*** |
| Total CA | - | 101(39)/155(61) | 0.73 (0.53-1.02) | 0.067 | 0.080 | 24(35)/45(65) | 0.95 (0.51-1.78) | 0.872 | 0.939 | 77(41)/110(59) | 0.67 (0.45-0.99) | **0.042** | **0.041** |
|  | + | 122(32)/255(68) |  |  |  | 39(34)/77(66) |  |  |  | 83(32)/178(68) |  |  |  |
| Gastric CA | - | 202(36)/363(64) | 0.82 (0.48-1.41) | 0.476 | 0.438 | 51(33)/104(67) | 1.36 (0.61-3.04) | 0.454 | 0.577 | 151(37)/259(63) | 0.55 (0.25-1.20) | 0.133 | 0.126 |
|  | + | 21(31)/46(69) |  |  |  | 12(40)/18(60) |  |  |  | 9(24)/28(76) |  |  |  |
| Colorectal CA | - | 203(35)/369(65) | 0.91 (0.52-1.60) | 0.740 | 0.672 | 58(34)/113(66) | 1.08 (0.35-3.38) | 0.892 | 0.989 | 145(36)/256(64) | 0.85 (0.45-1.64) | 0.634 | 0.613 |
|  | + | 20(33)/40(67) |  |  |  | 5(36)/9(64) |  |  |  | 15(33)/31(67) |  |  |  |
| Lung CA | - | 208(35)/393(65) | 1.89 (0.91-3.94) | 0.090 | 0.097 | 58(33)/119(67) | 3.42 (0.79-14.80) | 0.100 | 0.125 | 150(35)/274(65) | 1.52 (0.64-3.61) | 0.340 | 0.338 |
|  | + | 15(50)/15(50) |  |  |  | 5(63)/3(37) |  |  |  | 10(45)/12(55) |  |  |  |
| Blood CA | - | 204(36)/365(64) | 0.77 (0.44-1.36) | 0.371 | 0.416 | 55(34)/105(66) | 0.90 (0.37-2.21) | 0.816 | 0.967 | 149(36)/260(64) | 0.71 (0.34-1.47) | 0.359 | 0.343 |
|  | + | 19(30)/44(70) |  |  |  | 8(32)/17(68) |  |  |  | 11(29)/27(71) |  |  |  |
| Alcohol | - | 189(35)/348(65) | 1.04 (0.66-1.65) | 0.855 |  | 42(34)/81(66) | 1.01 (0.53-1.93) | 0.970 |  | 147(36)/267(64) | 1.18 (0.57-2.44) | 0.654 |  |
|  | + | 34(36)/60(64) |  |  |  | 21(34)/40(66) |  |  |  | 13(39)/20(61) |  |  |  |

CA, cancer; WW, wild-type (reference) ; WD, heterozygote; DD, whole-gene deletion;－(cancer-free); + (cancer-bearing).

* Represents adjusted by age, gender and drinking.

** Represents adjusted by age and drinking
